# A mixed treatment comparison of Erlotinib vs. placebo and Erlotinib vs. Geftinib using Bayesian approaches

**DOI:** 10.1186/1745-6215-14-S1-P119

**Published:** 2013-11-29

**Authors:** Iftekhar Khan, Gianluca Baio, Saira Ahmed

**Affiliations:** 1 University College London, London, UK

## Background

Erlotinib and Gefitinib have proven efficacy in patients with non small cell lung cancer (NSCLC). However, evidence for a direct comparison between the two treatments does not exist. We conducted a mixed treatments comparison (MTC), so that an indirect comparison of Erlotinib vs. Gefitinib can be made and a formal economic evaluation can be consequently undertaken.

## Methods

Evidence was synthesized from published randomised controlled phase III trials , including recent evidence for Erlotinib from a trial sponsored by the CRUK & UCL Cancer Trials Centre comparing Erlotinib with Placebo. The effects of Erlotinib and Gefitinib on overall survival (OS) and progression-free survival (PFS) were compared using a MTC method with Placebo as the common comparator. A Bayesian approach was used to carry out the MTC. A further economic evaluation was undertaken under a Bayesian framework.

## Results

Seven randomised controlled trials were included. Both interventions offered improvements in OS and PFS compared with placebo. Using MTC analysis, the OS hazard ratio (HR) for Erlotinib vs. Placebo was 0.83; 95% credible interval (CI) of (0.31 , 2.38); for PFS HR=0.81; 95% CI (0.12,5.65); for Gefitinib vs Placebo: OS HR= 0.86; 95% CI (0.27, 2.54); PFS HR was 0.42; 95% CI (0.06,2.79); OS HR for Erlotinib vs Gefitinib was 0.98; 95% CI (0.24,4.58) and for PFS was 1.84; 95% CI (0.14, 31.2).

## Conclusion

Both Erlotinib and Geftinib show improved OS and PFS over Placebo on average. However, although Erlotinib and Gefitinib appear equivalent in terms of OS, Gefitinib appears to offer improved PFS. This is likely to improve HRQoL prior to progression and have an important impact on the cost per Quality Adjusted Life Year (QALY).

